# A model for crowdsourcing high-impact research questions for Castleman disease and other rare diseases

**DOI:** 10.1186/s13023-023-02678-6

**Published:** 2023-04-11

**Authors:** Ania Korsunska, Mileva Repasky, Mary Zuccato, David C. Fajgenbaum

**Affiliations:** 1 Syracuse University, 343 Hinds Hall, Syracuse, NY 13244 USA; 2 Castleman Disease Collaborative Network, 3535 Market Street, Suite 700, Philadelphia, PA 19104 USA

**Keywords:** Patient-centered research agenda, Crowdsourcing, Rare disease, Collaborative network, Castleman disease

## Abstract

**Background:**

There are approximately 10,000 rare diseases that affect around 30,000,000 individuals in the U.S.A., most of which do not have an FDA-approved treatment. This fact highlights the failure of traditional research approaches to overcome the unique challenges of developing rare disease treatments. The Castleman Disease Collaborative Network was founded in 2012 to advance research and treatments for Castleman disease, a rare and deadly disease that involves the immune system attacking the body’s vital organs for an unknown cause. It has spearheaded a novel strategy for advancing biomedical research, the Collaborative Network Approach. This approach consists of eight steps, one of which is to identify and prioritize high-impact research questions through crowdsourcing ideas from the entire community of stakeholders: patients, loved ones, physicians, and researchers. Rather than hoping that the right researcher will apply for the right research project at the right time, crowdsourcing high-priority research projects into a research strategy ensures that the most high-impact, patient-centered studies are prioritized. The Castleman Disease Collaborative Network launched an initiative in 2021 to systematically generate this list of community-directed studies to focus Castleman disease research efforts.

**Results:**

The Castleman Disease Collaborative Network was able to successfully create a patient-centered research agenda through engaging the entire community of stakeholders. The community contributed important questions about Castleman disease, which were prioritized and reviewed by our Scientific Advisory Board, and the result was a finalized list of studies that address these prioritized questions. We were also able to generate a best practices list which can serve as a model that can be utilized for other rare diseases.

**Conclusion:**

Creating a patient-centered research agenda through crowdsourcing research ideas from the community is one of the most important ways that the Castleman Disease Collaborative Network operationalizes its commitment to keeping patients at the center of research and we hope that by sharing these insights we can assist other rare disease organizations to pursue a patient-centric approach.

## Introduction

There are approximately 10,000 rare diseases that affect around 30,000,000 individuals in the U.S. [1, 2]. Thus, rare diseases, though individually rare, when taken together constitute a substantial burden overall. Unfortunately, due to a variety of systemic factors, and despite significant investments of resources over the last several decades, most of these rare diseases still do not have any FDA-approved treatment; it is estimated that over 90% of rare diseases lack an effective treatment [3]. This fact highlights the failure of traditional research approaches to overcome the unique challenges faced by rare disease research.

**Fig. 1:**
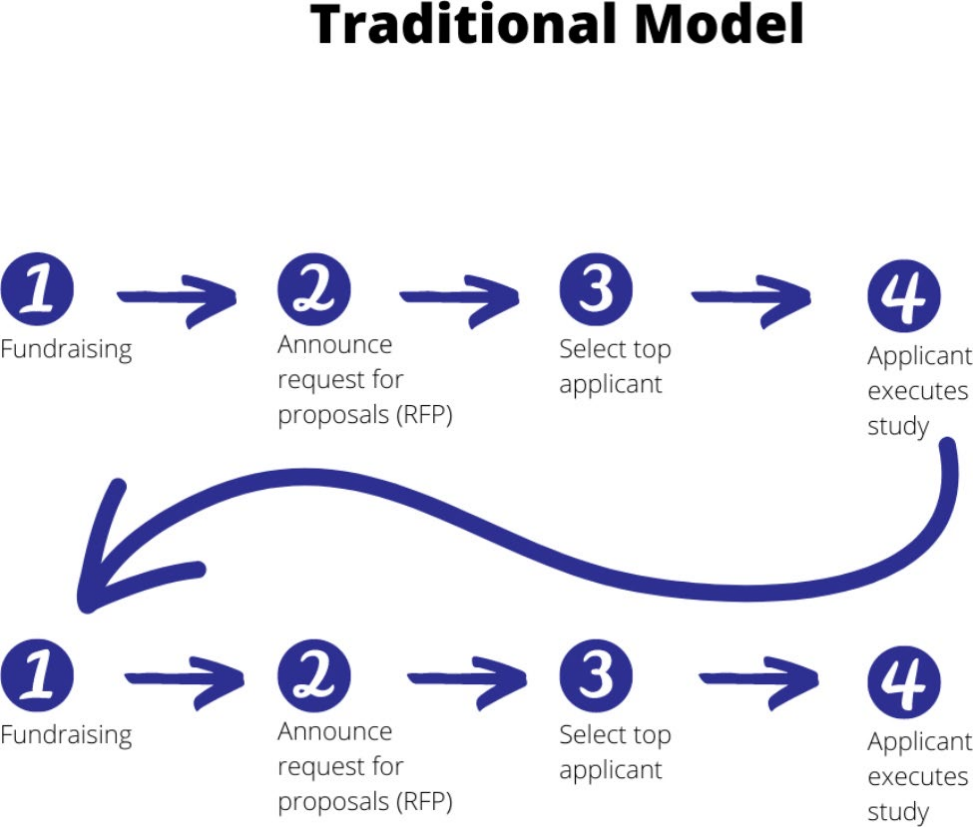
The traditional model of an organization supporting research

In the rare disease space, nonprofit organizations are often key players in supporting the patient community through outreach, advocacy, financial support, and treatment guidance as well as through advancing research and treatment discovery. The traditional way that rare disease nonprofit organizations support research involves raising funds, announcing a request for proposals (RFP) that invites researchers to submit a research proposal (including a research question, approach to answering the question, and budget) that addresses the area of research described in the RFP, and then awarding funds to the best proposal, selected by a panel of experts (Fig. 1).

This traditional model works well when there are a large number of applicants, and barriers to accessing key research material are low. Unfortunately, when the number of interested and qualified researchers in an area is limited, as is the case in the rare disease space, it is less probable that a single proposal will both address a high-impact question and be submitted by the most qualified researcher. Great research ideas are never pursued if they are not conceived by a researcher or if a researcher never applies for the grant to do the work. Therefore, rare disease organizations must hope that the right researcher with the right skill set and access to the requisite biospecimens will conceive of the right study and apply for funding. Furthermore, each submission is developed independently and judged independently from the others, preventing a coordinated, disease-wide plan from emerging. As such, research foundations’ research project portfolios are often fragmented and uncoordinated. The RFP process leads to competition between researchers and limits collaboration, where samples and research ideas become assets for grant applications. This is particularly problematic for rare disease research as patient samples are inherently scarce. Without a sufficient number of samples, meaningful insights cannot be made. Another critical problem with the traditional model is that patients, the very people who share the lived experience and are in need of treatments and solutions, are not involved in crafting or molding research priorities.

**Fig. 2:**
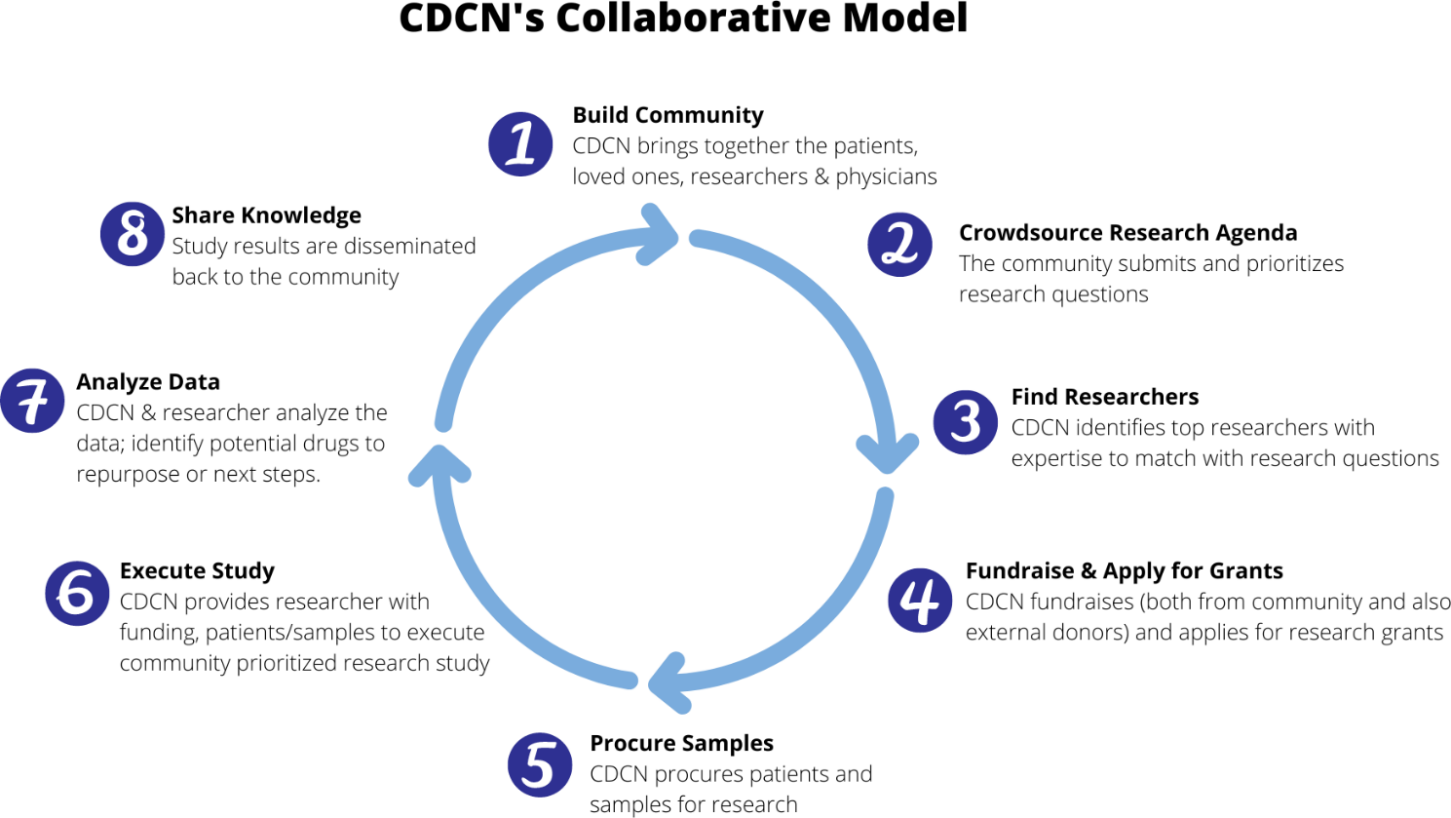
CDCN’s eight step Collaborative Network Approach [4]

Due to the suboptimal research infrastructure and treatment options for Castleman disease, the Castleman Disease Collaborative Network (CDCN) was founded in 2012. As a part of its mission to improve survival for all patients with Castleman disease, the CDCN developed a novel approach to advancing biomedical research, called the Collaborative Network Approach [4]. To overcome the limitations of the traditional research model, the CDCN’s Collaborative Network Approach leverages and integrates the entire community of stakeholders — patients, loved ones, physicians and researchers — to identify and prioritize high-impact research questions through eight steps (Fig. 2).

First, this approach starts by building an engaged community of patients, loved ones, physicians, and researchers through a contact registry, community events, an internal communications platform, and social media outreach (Step 1). Then, this community comes together to participate in creating the research agenda (Step 2). This is done through a crowdsourcing approach to make sure that the research priorities not only are informed by the latest developments in biomedical research, but also are led by the needs of patients and loved ones. Once the research agenda is created, the CDCN identifies the most qualified researchers to conduct these studies based on the skill set needed for the project (Step 3). The CDCN is continuously applying for grants and engaging in fundraising initiatives so that when the research questions are prioritized and researchers identified, there will be sufficient funding to support the work (Step 4). Patients are also empowered to keep engaged in the research process through fundraising and providing their samples and clinical data. The CDCN works on securing and storing these biospecimens and clinical data (Step 5). Once the researchers are identified, the CDCN provides them with the necessary funding, data and samples needed to complete the work (Step 6). The CDCN works closely with the researchers to analyze the data and take vital insights from the research findings (Step 7). Finally, the CDCN shares the findings and knowledge with the community (Step 8). This eight step Collaborative Network Approach is a way to democratize the research process, identify the most clinically relevant questions posed by the very community which will be directly impacted by the answers. This approach encourages researchers to think creatively in order to answer these questions and suggest lines of inquiry that might be outside of the traditional way of thinking, which is often constrained by grant proposal requirements [4].

The Collaborative Network Approach has led to significant progress for the Castleman disease field between 2012 and now, including discovering the first-ever FDA-approved treatment, first new therapeutic approach in 25 years, first-ever diagnostic criteria, and first-ever treatment guidelines. In 2019, the CDCN partnered with the Chan Zuckerberg Initiative to develop and optimize a communications platform to facilitate improved crowdsourcing of research ideas. In 2021, the CDCN launched this platform to crowdsource and prioritize questions from the entire Castleman disease community and guide the next generation of research studies [6], which we will describe herein.

## Methods

**Fig. 3:**
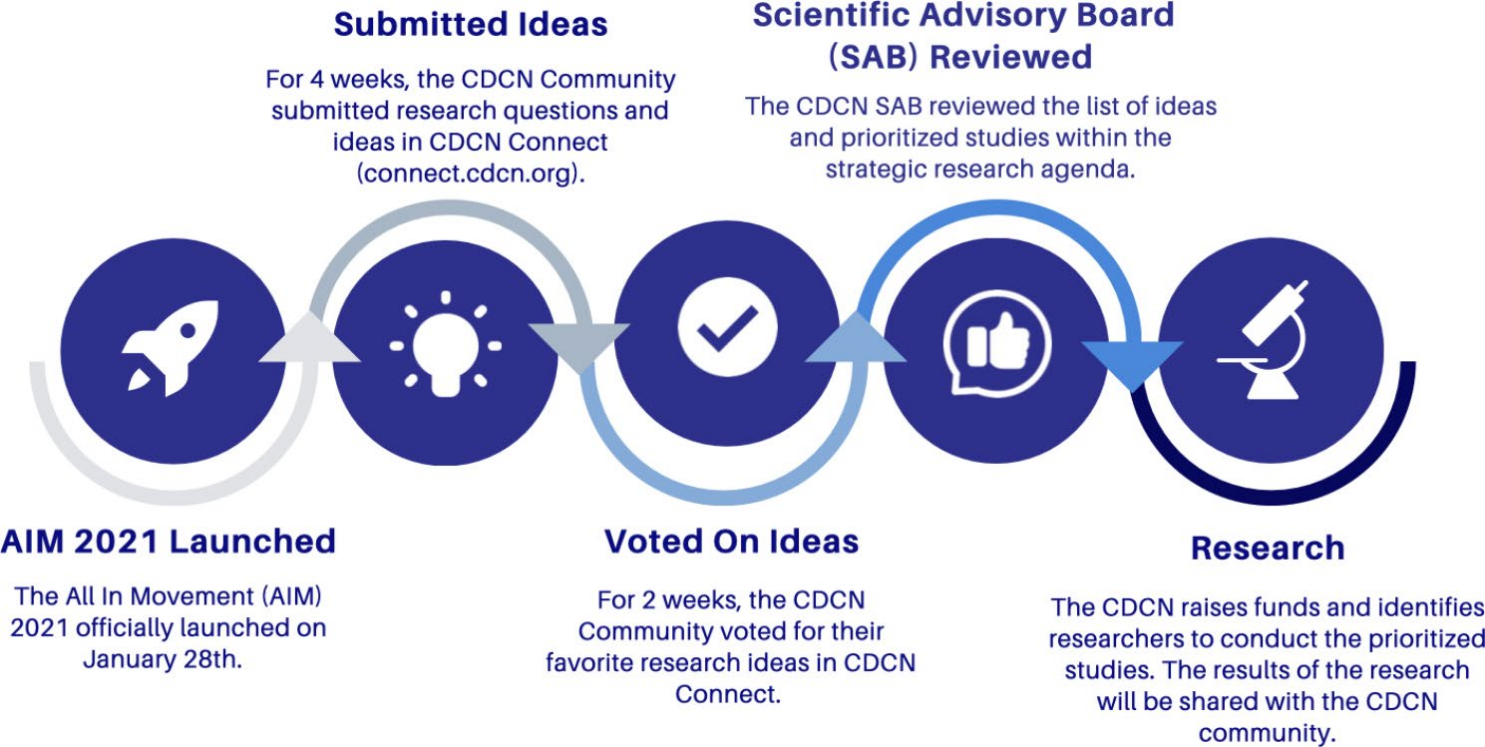
CDCN’s five-phase approach to crowdsourcing research ideas

The CDCN team established a five-phase plan for crowdsourcing ideas from the community to generate a list of community-directed, high priority studies to focus research efforts (Fig. 3).

By utilizing this five-phase approach, the CDCN team set a goal of receiving at least fifty questions about Castleman disease generated by the Castleman disease community. The community consists of Castleman disease patients, loved ones (parents, spouses, friends, siblings, etc. of a rare disease patient; this does not include paid care staff), physicians who treat Castleman disease, and researchers who study Castleman disease. The submitted questions would then be reviewed by the CDCN Scientific Advisory Board (SAB) to plan studies that address these prioritized questions. This approach shares some similarities with one taken by James Lind Alliance in the United Kingdom, which used a similar crowdsourcing and prioritization process to identify the top 10 research priorities for rare musculoskeletal diseases [5]. One significant difference is the types of stakeholders included in the process: the James Lind Alliance utilized input from patients, carers and healthcare professionals, while the CDCN also included researchers in both our crowdsourcing and prioritization process, as well as the involvement of the CDCN’s SAB in prioritizing the research studies, discussing funding allocation and working to locate researchers to pursue these studies, as outlined in CDCN’s Collaborative Network Model [4].

**Fig. 4:**
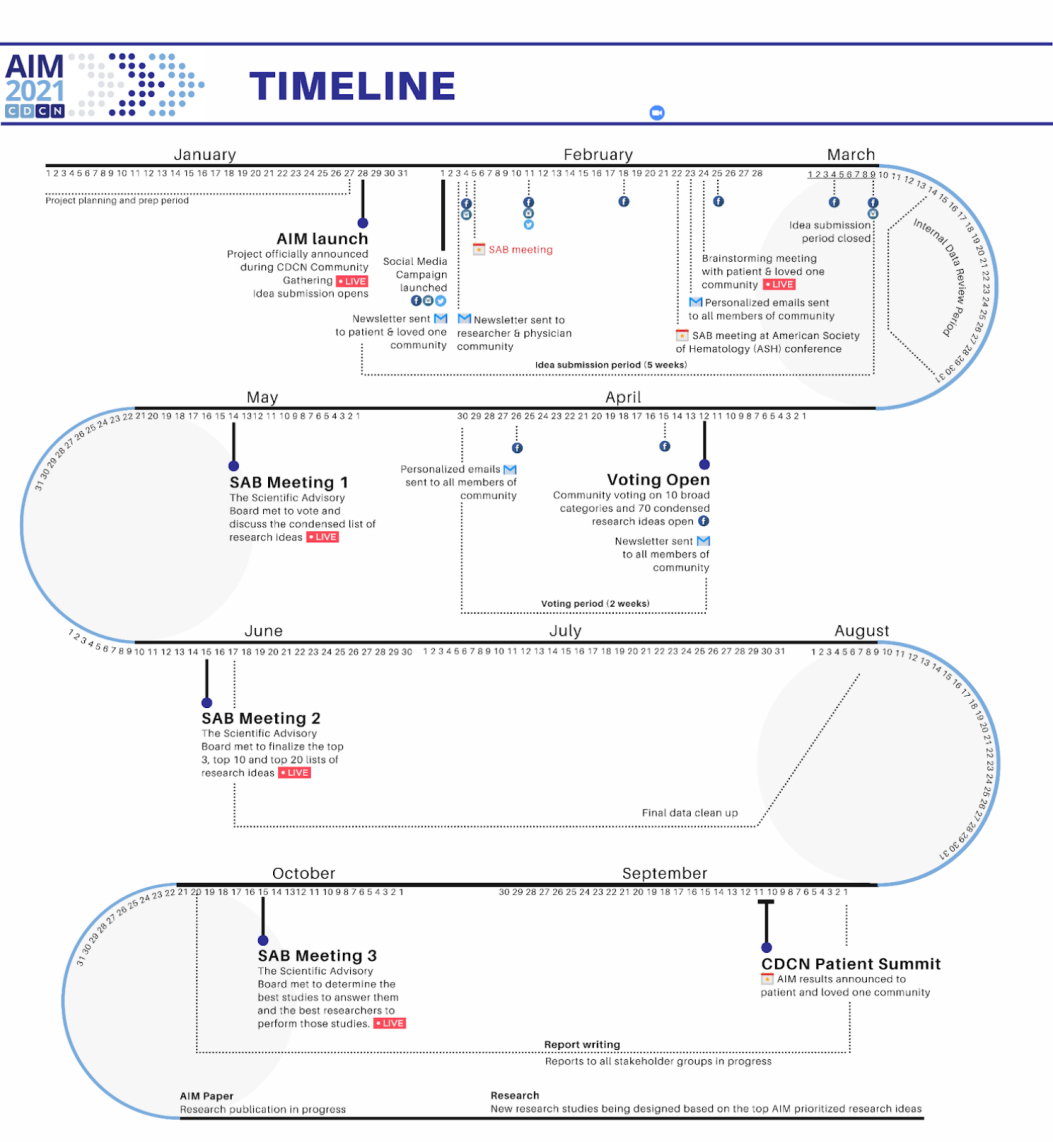
Timeline of the crowdsourcing process

A detailed timeline of the crowdsourcing process is outlined in Fig. 4, including all phases, meetings and community events that took place to help facilitate the process.

### Preparation: user-experience interviews

Before launching the initiative, a series of short interviews were conducted with patients and loved ones from the CDCN community. The main goals of these interviews were to establish current levels of engagement with the platform which we would utilize for the crowdsourcing initiative, understand the levels of usability of the platform, and anticipate and implement safeguards to avoid potential problems. The interviews were conducted via 1:1 video conference calls. The questions asked during the interviews were based on usability, efficiency, and case use scenarios.

### Phase 1: Launch/Awareness-raising

Through social media posts, community gatherings, direct email and live video sessions, the team raised awareness among patients, loved ones, physicians, and researchers from the CDCN community about the initiative and invited them to participate.

### Phase 2: idea submission & synthesis

Community members were invited to share their research ideas over a period of five weeks. The team set up two separate spaces on our online platform: one for researchers and physicians to contribute research ideas, and one for patients and loved ones. Therefore, both communities were able to submit and comment on ideas only within their own space. Though both communities were asked to submit ideas, they were asked slightly different prompting questions.

Patients and loved ones were asked: “What are the most impactful questions that you have about Castleman disease?”, while researchers and physicians were asked: “What questions would be most impactful to patients if they were answered through research?” All participants were offered multiple avenues to contribute research questions and ideas: email, social media, posts on the internal CDCN platform and in live video conference community meetings.

**Fig. 5:**
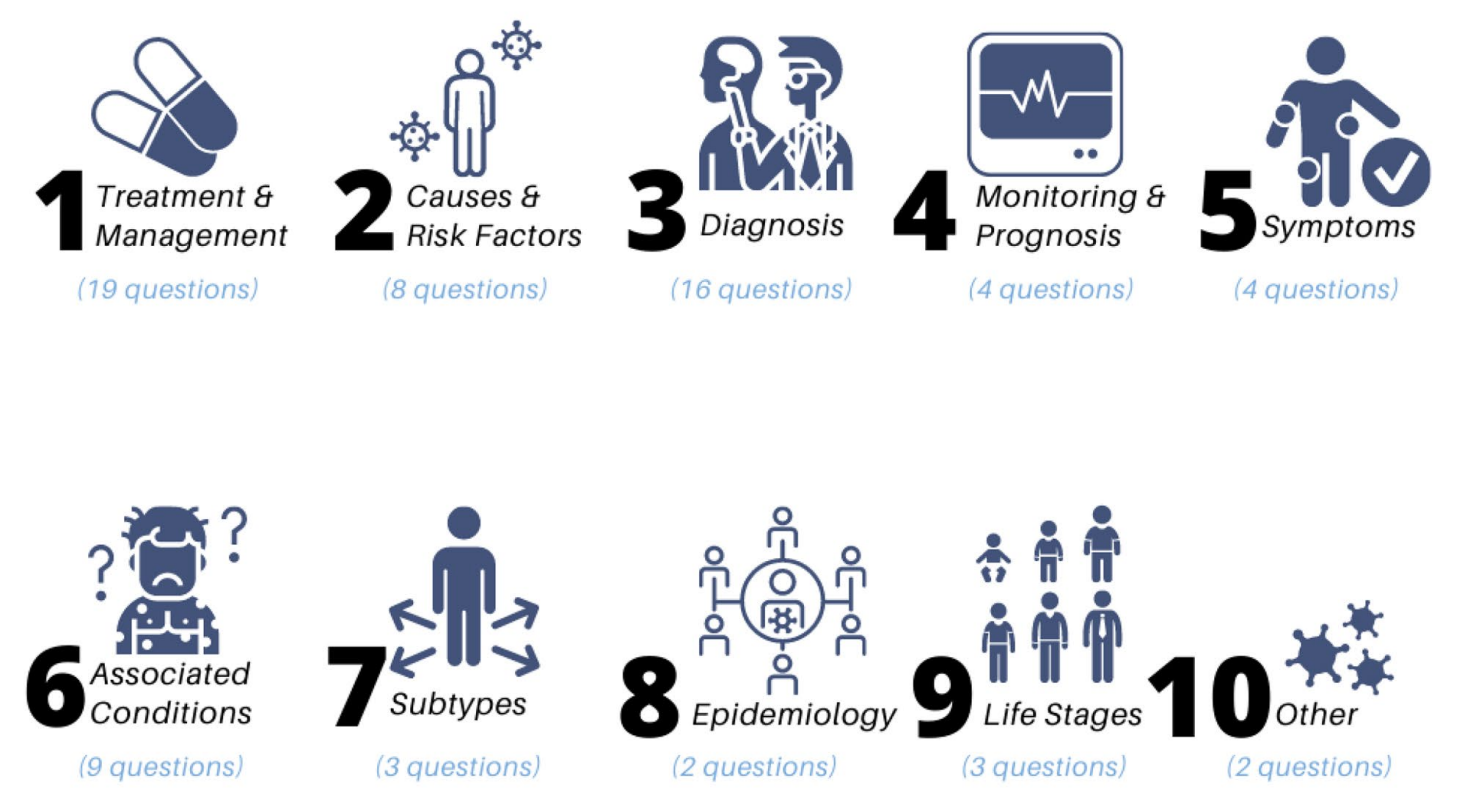
Ten broad research categories identified through idea synthesis

Once the idea collection was completed, the CDCN team set out to synthesize the 155 submissions by condensing and organizing them in a way that would make it practical for the community to review and vote for the ones they cared most about, so those could be prioritized for future research. One of the outcomes of this synthesis was ten categories which reflected the topics of the questions **(**Fig. 5**)**.

This idea compilation and synthesis was accomplished through a four-step process. First, all submitted ideas were compiled in a spreadsheet. Next, the CDCN team conducted minor data cleaning and organizing: fixed spelling errors, separated out submissions with multiple questions, and removed personal information. Then, the questions were sorted into the 10 broad categories **(**Fig. 5**).** Finally, we combined similar entries or those with overlapping concepts; ideas were removed if they already had an answer based on prior research or were too specific to an individual patient’s case (these were to be addressed on a case by case basis with the individuals who submitted). The result was 70 research ideas.

### Phase 3: Community Voting & Synthesis

The next step was to prioritize the various research ideas. This was accomplished via a poll in which members of the entire community together (patients, loved ones, physicians, and researchers) were invited to vote on ideas together. The poll was split into two parts. In the first, the community was asked to rank the ten broad categories **(**Fig. 5**)** by importance (ranking from 1 to 10, with 1 being the highest importance), and in the second, they were asked to vote for their top choice of research ideas within each of the ten categories. This two-step voting process allowed the community to both rank what general areas of research they felt were most important for research to focus on and rank the most important questions for them within each broad category.

### Phase 4: Scientific Advisory Board (SAB) Review & final ranking

**Fig. 6:**
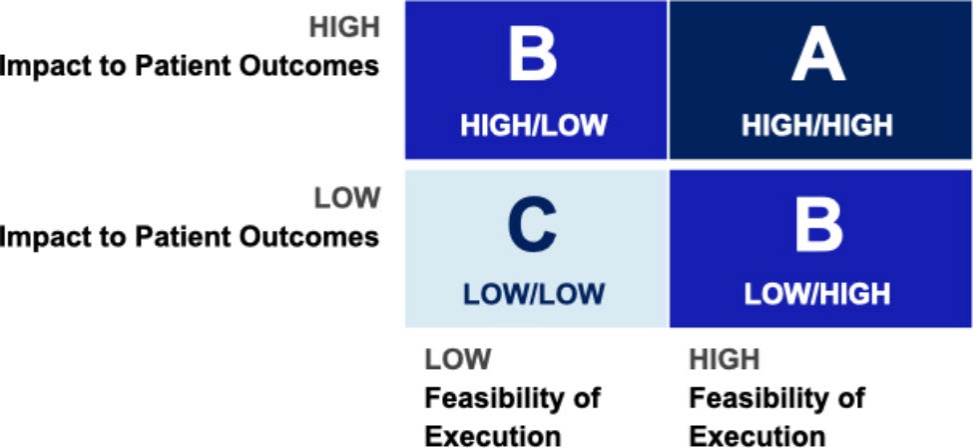
Grading guidance given to members of the Scientific Advisory Board (SAB)

Once the ideas were finalized and prioritized by the community, the CDCN SAB reviewed these priorities to determine a final ranking. In order to facilitate an effective SAB discussion, the team generated reports from the voting platform to help to streamline the SAB discussion. These reports included rankings of responses for categories (ranked 1 to 10) and specific ideas (ideas ranked 1–70 from ones that had the most votes to the least votes) by audience type (patients/loved ones or physicians/researchers) and total number of votes for each idea. The SAB members were asked to review the full list of ideas and the community voting data and assign a letter grade (A through C) for each idea, grading both on the impact on patient outcomes and feasibility of execution (Fig. 6).

Based on the likelihood of having a meaningful patient impact and feasibility of conducting the research, the SAB selected the top twenty, and then sorted them into three tiers: top 3, top 10 and top 20. Opinions of patients and loved ones were considered throughout the entire process of idea submission and prioritization, including SAB review, as we generated reports from the voting platform to help to the SAB see what the community prioritized as most important.

### Phase 5: Research & Next Steps

The finalized list of community-prioritized research questions was shared back with the community. This prompted the next stage of the Collaborative Network Approach: identifying the best researchers to take on the studies and securing funding for the new research agenda. The CDCN will allocate funding to the top-ranking ideas and, if necessary, raise additional funding from donors for priority studies. The CDCN’s Research Pipeline will be updated on an ongoing basis to track the progress of each study. As the Collaborative Network Approach outlines, the research study findings will be translated into patient impact as soon as possible through searching for potential repurposed drugs based on the results of the studies. The community will continue to improve its crowdsourcing abilities because the CDCN will disseminate knowledge about its findings and the prioritization process will be repeated every few years. There is already a paper in process focused on the experiences of UCD patients following lymph node excision that was inspired by this project.

The final step in this initiative was gathering feedback from the participants in order to identify opportunities for improvement and iterate upon the process for the future. This follow up was in the form of a short survey to all patients and loved ones via email, regardless of whether they participated or not. For those who participated in the initiative, we asked them the following questions:

- How did you participate? (Check all that apply)
  - Liked and shared social media
  - Submitted research questions/ideas
  - Voted on prioritization
- Why did you choose to participate this year? (Open text)
- How would you rate the clarity and effectiveness of communication around this opportunity and the process to participate? (Scale of 1–5)
- How likely are you to recommend that other patients engage in this initiative in the future? (Scale of 1–5)
- How would you rate the ease of using the platform? (Scale of 1–5)
- What specific aspects of the platform contributed to your rating? (Open text)
- What would make participation for you in the future? (Open text)

For those who did not participate, we asked the following questions:

- Why did you not participate in the initiative this year? (Open text)
- How likely are you to participate in this initiative in the future? (Scale 1–5)
- How would you rate the clarity and effectiveness of communication around this opportunity and the process to participate? (Scale 1–5)
- What would make participation easier for you in the future? (Open text)

## Results

The crowdsourcing initiative met its primary objective to generate a list of community-directed, high priority studies to focus Castleman disease research efforts [6]. There was active community participation across patients, loved ones, physicians, researchers. Though originally the CDCN set a goal of 50 ideas, the final count was 155 ideas submitted, with 35 patients and loved ones and 10 physicians and researchers having participated. 8 of the 10 physicians and researchers who submitted ideas are members of the CDCN SAB. In the voting phase, we had double the participation compared to the idea submission phase: 70 individuals from the community participated, casting a total of 2,641 votes. Out of the total 30 members of the CDCN SAB, 21 participated in either voting or idea submission, or both. The fact that there were more patients and loved ones who participated, compared to researchers and physicians, was consistent with our idea that the research agenda should be patient-centered and that research priorities not only are informed by the latest developments in biomedical research, but also are led by patients’ and loved ones’ needs. It is also worth mentioning that the CDCN has pioneered a patient-powered natural history study design called ACCELERATE and will be utilizing the research ideas prioritized through this project in order to design patient-centered studies in ACCELERATE in the future [7].

Importantly, we found strong alignment of priorities between the patient and loved one community and the physician and researcher community, throughout the various stages of the prioritization process. The various stakeholders found similar topics to be important and prioritized those over others. These can be categorized into four top themes:

1) *Treatment and Management*: Treatments to help patients feel better or be in remission.

2) *Cases and Risk Factors*: Risks of being on medications long term.

3) *Diagnosis*: Improving diagnosis.

4) *Monitoring and Prognosis*: Steps to ensure patients stay healthy and on top of their disease.

Overall, the entire Castleman disease community successfully contributed important questions about Castleman disease, which were then prioritized by the CDCN SAB, and finalized into a list of studies that address these questions. These studies were integrated into CDCN’s research pipeline and are currently ongoing [8].

### User experience interviews

The team conducted interview sessions with six members of the community: three patients and three loved ones. The feedback obtained from the preliminary user-experience interviews was used to develop a short video tutorial on how to engage with the platform during the idea submission and voting process. In addition, changes were implemented to the actual layout and functionality of the platform to enable it to be more user-friendly (i.e., eliminated the character limits, edited the toolbar, adjusted language in the prompts, added visuals, etc.). These interviews also helped to select which of the several voting options available on the platform was the most user-friendly and would work best to be able to accomplish our project goals.

### Follow up Survey

**Fig. 7:**
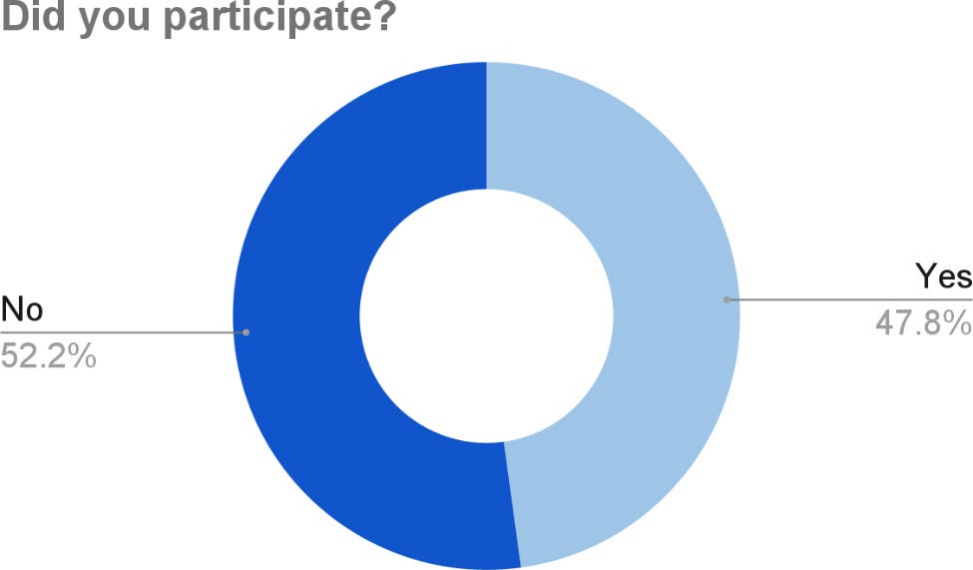
Participation among the follow up survey participants

The CDCN conducted a short follow up survey to gather feedback from the community in order to identify opportunities for improvement. In total, 23 people participated in the survey. Out of this total, 47.8% (11 people) participated in the crowdsourcing initiative (Fig. 7). The participants communicated that they felt strongly about wanting to participate so they could help find a cure and help with research efforts.

**Fig. 8:**
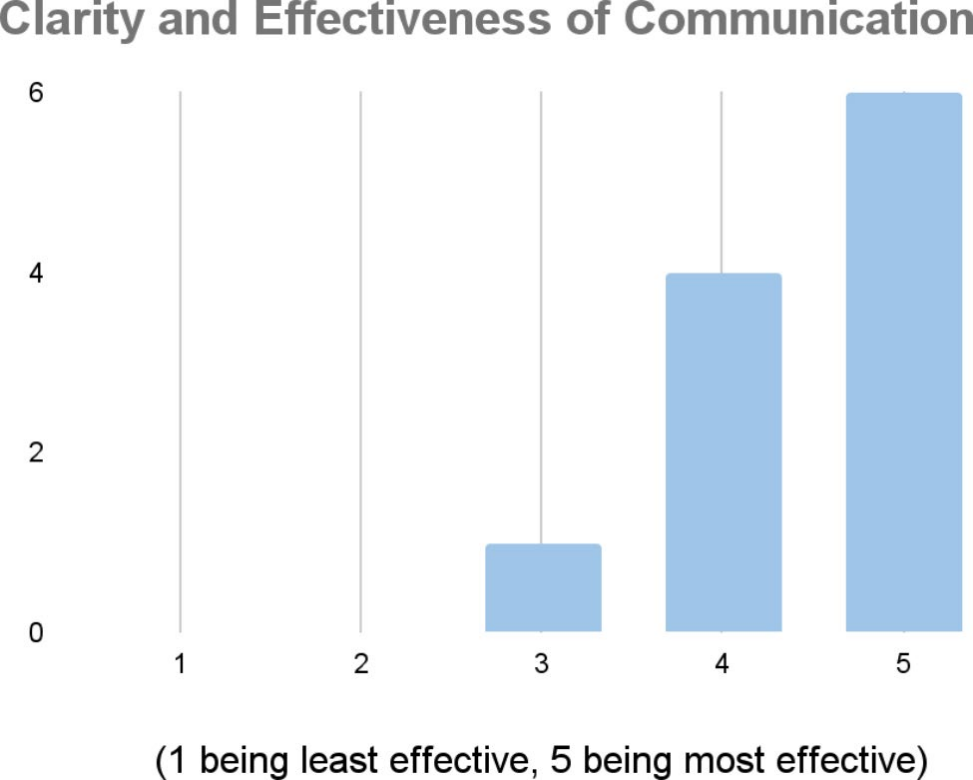
Ranking of the clarity and effectiveness of communication around the initiative

The people who did participate overall felt that the communication regarding the process was clear and effective (Fig. 8). When participants were asked about the ease of using the platform, there were mixed reviews. The participants reported that the platform was user-friendly and easy to use, but they felt that it wasn’t utilized enough by the community, and they were not made aware when something new was posted.

The areas for improvement that were highlighted were around lack of understanding of medical terms (providing definitions for categories) and improving the directions for the voting phase (e.g., communicating the scale of 1–10 so that it is understood what is highest/lowest). Other areas of improvement include removing various barriers to participation, such as issues with platform access, link expiration and login issues.

**Fig. 9:**
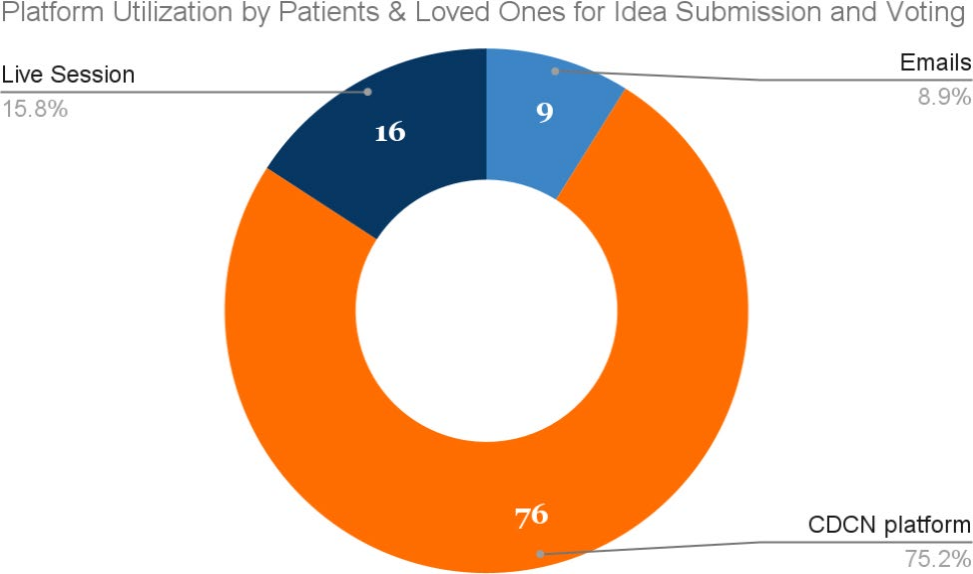
Participation methods among patients and loved ones

**Fig. 10:**
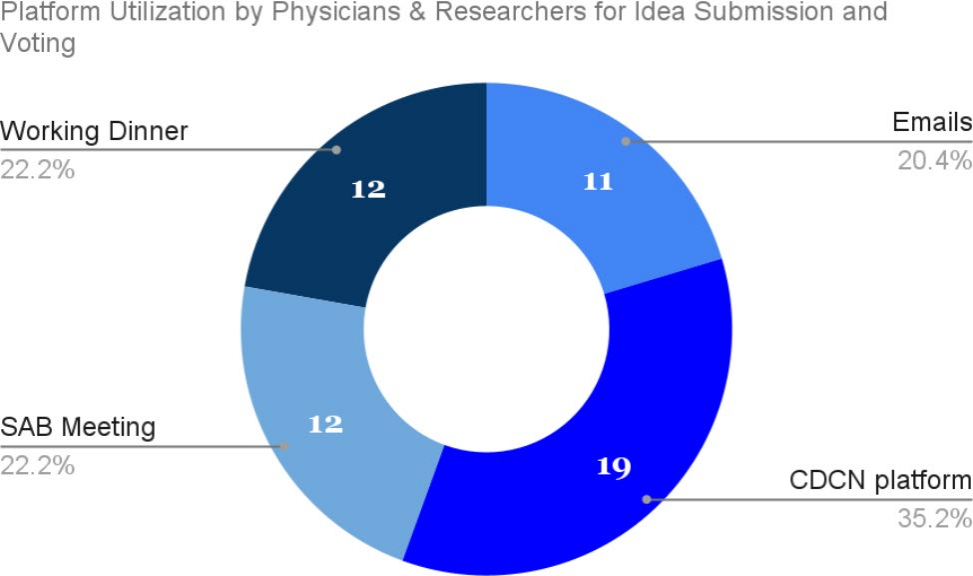
Participation methods among physicians and researchers

Despite some barriers to participation for some users, the CDCN communications platform was successfully utilized by the CDCN community. Though, it’s important to note that the patient and loved one community utilized the platform more than the physician and researcher community, who preferred live meetings and emails: 75% of all participation from patients and loved ones took place on the platform, and not social media or direct email, while only 35% of the physician and researcher engagement took place via the platform **(**Figs. 9 and 10**)**.

### Best Practices

As a result of all the community feedback, team reflection and the overall experience of the crowdsourcing initiative, we can provide the following list of best practices **(**Table 1**)**, which we believe can lead other rare disease organizations who may wish to utilize this approach to create a patient-centered research agenda.

**Table 1:** Best Practices

1. Create a dedicated team with a mix of skills to execute the various aspects of the initiative, including a dedicated project manager.
2. Manage a single, shared communications documents for all team members to edit and review newsletter text and social media posts.
3. Conduct user experience research as needed to ensure smooth experience with any platforms or tools being utilized.
4. Remove as many barriers to participation as possible for all community members. Make platform use and communication as simple and easy as possible through written and video instructions and be available to help troubleshoot issues.
5. Run a two-phase process with separate crowdsourcing and voting/prioritization phases for physicians/researchers and patient/loved ones.
6. Use personal, direct communication via email, phone, text, social media DM, to community members to yield the best results for participation.
7. In the idea submission phase, give participants examples for what a research question is, how to turn a general topic into an idea.
8. The idea review and consolidation process is labor-intensive and complex. A certain skillset and comfort with data analysis is required in order to categorize and group ideas accurately. There are many ways to present the groups of ideas to the SAB and ask them to rank and/or prioritize (i.e. rank by feasibility), and this is best decided on ahead of any data analysis.
9. Maximize productivity among the SAB during live meetings: a. To better prioritize what questions to discuss during SAB meetings, include a metric that indicates which questions were rated highly by some members of the SAB and poorly by others. These metrics help to generate rich discussion on the more “controversial” questions. b. Display votes entered early, and update live during the session. c. Consider creating a SAB sub-committee for the final review to determine the top lists.
10. Gain as much feedback from the community as possible to improve for future iterations.

## Discussion

Research must build on existing knowledge and address gaps in understanding. Traditionally, what research is pursued is decided by researchers, based on literature review and expertise. But though researchers may be experts in their field, they lack the real-world patient experience of having a rare disease. Patients and researchers may have different priorities when it comes to evaluating the effectiveness of a treatment or therapy. While researchers may focus on measures such as overall survival rates, patients may place more importance on factors that affect their quality of life. For example, patients with chronic rare diseases may be more concerned with the impact of their disease or treatment on factors such as fertility, cognitive development, or pain management. Additionally, patients may prioritize more convenient drug delivery methods, such as ones that do not require regular trips to a hospital. It’s important to understand and take into account these patient-centered outcomes when evaluating the effectiveness of a treatment or therapy. One potential solution to make sure these preferences are taken into account is by crowdsourcing research ideas, as it allows for public participation in setting research agendas and promotes collaboration and democratization of science [9, 10].

In recent years, there has been a growing emphasis on involving other stakeholders in the research process, particularly in the field of medicine [11–14]. Crowdsourcing approaches have already been used as a part of data sharing and collaboration [15, 16], development of research questions and data analysis [4, 9, 17] and the development of drugs [18–20]. There is a growing consensus in the industry that without integrating the voices of all the patient population, it becomes “impossible to identify the most clinically meaningful questions and research approaches to answering them” [4].

### Limitations

Although the results of our study indicate success in terms of creating a patient-centered research agenda through crowdsourcing, it is important to recognize the limitations of the study. Specifically, the findings may not be easily generalizable to other rare disease communities due to the unique nature of relationships between patients, loved ones, physicians, and researchers in each community. Additionally, the tools we used for this initiative have been effective, but there are opportunities for improvement and alternative tools that may be better suited for crowdsourcing research ideas from the community. Furthermore, while crowdsourcing from the community has proven to be an effective method for creating a patient-centered research agenda in this case, there may be other approaches that are equally successful in gathering these insights that we may have overlooked.

## Conclusion

Crowdsourcing research questions is one of the most important ways that the CDCN operationalizes its commitment to keeping patients at the center of research. Additionally, the Collaborative Network Approach makes bi-directional sharing of insights possible and keeps the research patient centered [4]. The CDCN facilitates communication from patients and loved ones to physicians and researchers, so that the research being conducted matches what is most important to patients. The CDCN also facilitates communication from researchers and physicians back to the patient and loved one community, so that patients are well-informed of the state of research of their disease and know areas that have limited understanding. In this paper, we share our findings and best practices from the initiative to serve as a resource for other rare disease organizations seeking to address research and treatment discovery for their diseases.

## Data Availability

The CDCN’s Research Pipeline (https://cdcn.org/physicians-researchers/research-pipeline) will be updated ongoing to track the progress of each study which was prioritized with this initiative.

## List of abbreviations

CDCN: Castleman Disease Collaborative Network
RFP: Request for proposals
SAB: Scientific Advisory Board
UCD: Unicentric Castleman disease
